# Concurrent Treatment of Opioid and Tobacco Use Disorder in a Telemedicine Clinic: Case Report of Breaking Through Barriers

**DOI:** 10.2196/72872

**Published:** 2025-05-15

**Authors:** Darcy Michero, Laura Monico, Peyton Pielsticker, Larissa J Mooney, Suzette Glasner

**Affiliations:** 1 Department of Clinical Affairs Digital Therapeutics New York, NY United States; 2 Department of Psychiatry University of California Los Angeles, CA United States

**Keywords:** opioid use disorder, tobacco use disorder, buprenorphine, nicotine replacement therapy, case report

## Abstract

There are a myriad of obstacles faced by individuals who take medication for opioid use disorder, including high out-of-pocket costs, stigma, logistical challenges associated with traveling to treatment facilities, and negative experiences with treatment providers. The proliferation of illicit fentanyl use and associated overdose risk underscores the urgent need to broaden access to lifesaving pharmacotherapy for opioid use disorder. This case report describes the safe and effective induction of a 32-year-old male onto sublingual buprenorphine in a digital care setting, with integrated, counselor-facilitated psychosocial treatment delivered via a smartphone app targeting relapse prevention. The digital care modality enabled this patient to overcome 2 major treatment barriers: limited access to evidence-based treatment given his residence in a rural area, and heightened overdose risk given his use of fentanyl, a potent synthetic opioid. In addition, we describe tobacco cessation treatment delivered digitally concurrently with opioid use disorder care, combining pharmacotherapy and behavioral skills training. Given the disproportionately high rate of co-occurring opioid use disorder and tobacco use and associated mortality rates, the effective treatment of these conditions simultaneously in this case advances our knowledge of potentially efficacious treatment approaches for underserved individuals with opioid use disorder who present with polysubstance use.

**International Registered Report Identifier (IRRID):**

RR2-10.1177/20552076241258400

## Introduction

Buprenorphine, a medication used to treat opioid use disorder (OUD), reduces relapse and overdose risk and improves long-term treatment outcomes in individuals with opioid addiction. Studies have established the safety and efficacy of buprenorphine induction in both office-based and unobserved (ie, home) settings. Despite the wealth of evidence supporting their efficacy, only half or fewer of individuals diagnosed with OUD receive buprenorphine, and treatment dropout rates remain high, with an average treatment duration of less than 6 months [1]. Historically, treatment programs in rural areas were significantly less likely to offer buprenorphine than in urban areas because many practitioners lacked a Drug Enforcement Agency waiver to prescribe buprenorphine [2]. However, in efforts to boost rural accessibility to medications for opioid use disorder (MOUD), the United States Department of Health and Human Services updated its guidelines in 2021, which granted physicians expanded prescribing capabilities and reduced certification requirements [3]. Initially, though the elimination of the waiver requirement was expected to increase MOUD access, recent studies have highlighted that despite increases in buprenorphine prescribers, the number of patients who receive buprenorphine has not proportionally changed [4]. Furthermore, patients in rural areas may face unique challenges to buprenorphine access due to travel distance from specialists [5], and rural providers have cited concerns about misuse and diversion as barriers to prescribing buprenorphine [6]. Recent research indicates that a lack of adequate training resources for prescribers hinders the successful implementation of greater buprenorphine prescribing capacity [7,8]. As such, an urgent need remains to improve access to MOUD in nonmetropolitan areas.

Digital treatment modalities have been highlighted as a potential solution to overcome geographical barriers to accessing traditional care, including attending in-person visits, picking up medication from the pharmacy, or arranging transportation [9,10]. Recent evidence suggests that increasing access to buprenorphine via telemedicine facilitates treatment engagement and reduces overdoses [11]. Although some providers have fears of diversion or risk associated with an unobserved, home-based induction [12], both unobserved and observed buprenorphine inductions have been found to be safe and effective [13,14]. In light of the central role of potent synthetic opioids such as fentanyl in the opioid overdose crisis, telemedicine approaches have the potential to be transformative among individuals who are using fentanyl if implemented effectively. However, there is little guidance in the literature concerning telehealth-based buprenorphine induction for individuals who are actively using fentanyl and its analogs, for whom concerns about precipitated withdrawal, relapse, and overdose risk may pose barriers [15,16].

Polysubstance use is another critical public health problem among individuals with OUD, for whom nicotine co-use is highly prevalent, reported among over 80%-90% of the population [17]. In light of the disproportionately high mortality rate due to smoking-related illness among those with substance use disorders, coupled with findings that individuals with co-occurring OUD and tobacco use disorder face unique obstacles to smoking cessation, including limited access to treatment, low motivation to quit, and concerns about how cessation could impact their ability to manage opioid withdrawal symptoms (eg, negative affect) without relapsing [18], novel and scalable approaches to addressing nicotine use in this population are urgently needed.

Although evidence-based pharmacotherapies such as nicotine replacement therapy (NRT) increase cessation rates among those with OUD, research to inform digital care models addressing opioid and nicotine use concurrently is lacking [19]. This case report discusses the clinical characteristics, course, and outcomes of an individual with co-occurring OUD and tobacco use disorder who participated in a clinical trial evaluating the feasibility and preliminary efficacy of Pelago Opioid (Pel-O), developed by Digital Therapeutics, [20], a commercially available digital outpatient treatment program that combines MOUD, NRT, and evidence-based behavioral treatment for substance use disorders (SUDs; ie, cognitive behavioral therapy (CBT) and motivational enhancement therapy [MET]) in a digital care modality.

## Methods

### Overview

The patient was a 32-year-old Caucasian male with no significant past medical or surgical history. The patient was unemployed and lived with his significant other in a rural area in the southern Appalachian region. He had a family history of opioid addiction, indicating that he “watched (his) mom and dad use opioids” during his teenage years. The patient reported a history of an unspecified anxiety disorder, initiation of smoking a pack of cigarettes per day since he was 16 years old, and intermittent use of methamphetamine approximately once per month beginning at the age of 19 years. He reported initiating opioid use at 12 years of age when he experimented with recreational use of prescription opioids including Percocet and hydrocodone. At 14 years of age, he reported initiating the use of oxycodone 40 mg tablets regularly, and by the age of 16 years, he began to experience withdrawal symptoms in between episodes of opioid use. The patient continued to use illicit prescription opioids until the age of 28 years when he transitioned to using heroin and fentanyl exclusively via oral and intranasal routes of administration, a pattern he continued until 2 weeks before treatment initiation.

The patient’s treatment history includes 2 previous pharmacotherapy treatment episodes for OUD: (1) at 19 years of age, he obtained methadone from an opioid treatment program for 1 year, but discontinued treatment due to accessibility barriers including the cost associated with driving 200 miles per day to and from the clinic, as well as losing access to a driver’s license; and (2) at 26 years of age, buprenorphine was prescribed by an outpatient provider for 1 year but was also discontinued due to the same obstacles that impeded his first treatment episode (ie, transportation and cost).

Two weeks before enrolling in the Pel-O clinical trial, the patient’s opioid supplier was incarcerated, leading him to lose access to fentanyl and heroin. To avoid entering withdrawal, the patient purchased nonprescribed buprenorphine and reported illicit sublingual use of buprenorphine every other day, with 14 days of abstinence reported in the 30 days before treatment entry.

Pel-O is a digital SUD treatment approach with 2 essential components: (1) telemedicine-delivered medication management with a licensed nurse practitioner, doctor of medicine, or doctor of osteopathic medicine with expertise in addiction medicine for medication management, and behavioral therapy visits targeting relapse prevention with a licensed drug and alcohol counselor, using synchronous video visits via a smartphone app and (2) digital psychoeducation and evidence-based therapy skills training (eg, CBT and MET), delivered via the Pel-O and supplemented with counseling sessions (refer to Monico et al [20] for a detailed description of Pel-O).

### Ethical Considerations

The individual described in this case study was part of a larger clinical trial for which informed consent was provided for the provision of clinical care, as well as quantitative and qualitative interview-based data collection. The original informed consent as part of participation in the primary study allows for secondary analysis without additional consent. Oversight for this study was provided by the Ethical and Independent Review Services institutional review board. All study data are deidentified. Data for this case report were based both on quantitative data gathered over the course of the patient’s participation in the larger trial for which he was able to gain US $195 in gift cards as compensation, as well as a qualitative interview for which he was compensated with a US $50 gift card. Written informed consent was obtained from the patient for the use and publication of his clinical data for research following a detailed explanation of the objectives and protocol of the study.

At the patient’s first telehealth physician visit, he was assessed using a semistructured diagnostic interview based on the American Society of Addiction Medicine’s criteria for placement and treatment planning [21] and met *DSM-5* (*Diagnostic and Statistical Manual of Mental Disorders* [Fifth Edition]) criteria for severe OUD. He was determined to have a high risk for opioid relapse, necessitating outpatient care combining MOUD with behavioral skills training for relapse prevention. The patient subsequently attended eleven 30-minute video-based medical management appointments over a 5-month duration with his Pel-O physician; 8 visits over the first 3 months and 3 visits over months 4 and 5. Medical management visits included discussion of dose adjustments, side effects, and limited counseling as routinely provided to patients in outpatient settings. The patient’s initial prescription was issued to his local pharmacy to facilitate immediate buprenorphine initiation, whereas subsequent prescriptions were mailed directly to the patient’s residence according to his preferences, for convenience. Since the patient reported current use of nonprescribed buprenorphine at the time of treatment initiation, he was first prescribed sublingual buprenorphine with naloxone in the film formulation (hereafter, buprenorphine) at a dosage of 16 mg daily, with dosing evenly split twice daily. At the first and second follow-up visits, the patient reported continued cravings for opioids; accordingly, the prescribing physician adjusted his dosage to 20 mg, and subsequently to 24 mg, respectively, split evenly twice daily. The only notable side effect of buprenorphine reported by the patient was mild constipation that improved over time and with the aid of increased hydration and an over-the-counter stool softener. According to his report, the patient was prescribed hydroxyzine, quetiapine, and mirtazapine, each on an as-needed basis for sleep disturbance by an outside psychiatrist. At his final medication management follow-up in Pel-O, the patient reported that he was currently taking only mirtazapine regularly for sleep, though he did not specify the dosage.

The patient attended thirteen 45-minute video-based visits with a licensed drug and alcohol counselor over the course of 5 months of his Pel-O treatment participation. The counselor delivered manualized CBT and MET, with between-session asynchronous messaging through the chat function in the Pel-O smartphone app. Over the 6-month duration, the counselor sent 133 chat messages, and the patient sent 116 chat messages, demonstrating a high level of engagement in care. The patient also learned coping skills and exercises to help identify triggers pertaining to his cravings for both opioids and tobacco during these sessions.

Approximately 6 weeks after Pel-O treatment initiation, the patient expressed a desire to quit using tobacco, and after discussing nicotine replacement pharmacotherapy options, his Pel-O physician provider prescribed nicotine nasal spray. However, due to nasal mucosa irritation, the patient discontinued the use of the nasal spray and transitioned to alternative forms of NRT (ie, nicotine gum and patches). The Pel-O program provided 4 mg nicotine gum for as-needed use, as well as 21 mg and 14 mg nicotine replacement patches. After 4 weeks at 21 mg, the patient transitioned to the use of the 14 mg patches every 24 hours and subsequently titrated down to 7 mg per 24 hours. As instructed, the patient wore the patches over the course of 16 weeks. Concurrently, his CBT and MET counseling sessions incorporated his treatment objectives for smoking cessation to enable the use of cognitive and behavioral coping skills to prevent relapse to tobacco use.

After stabilization on buprenorphine, a total of 3 self-administered urine drug screens (UDS) were gathered at a monthly cadence. The patient was mailed a US Food and Drug Administration–approved 1-step test for the UDS and was asked to gather the sample privately while the clinician waited digitally for the patient to return to the camera to display the results. The UDS screened for the presence of opiates (heroin, morphine, and prescription drugs), fentanyl, amphetamines, benzodiazepines, methadone, cocaine, methamphetamine, and buprenorphine with a threshold >10 ng/mL considered indicative of medication adherence.

## Results

The patient’s first UDS gathered 8 weeks postinduction, was positive for opiates and negative for fentanyl. Other substance use detected included amphetamines, opiates, tetrahydrocannabinol (THC), and synthetic marijuana. Concurrently, the test result was positive for buprenorphine, indicating treatment adherence to the MOUD regimen. The second UDS, performed 26 days after the first, was negative for opiates and fentanyl and positive for buprenorphine. In terms of other substance use, the second UDS was positive for THC only. The third UDS performed a month later, was negative for opiates and fentanyl positive for buprenorphine, indicating continued adherence to MOUD care. The test also detected THC, while negative for all other substances. The patient’s depression, anxiety, craving for opioids, and cigarette use were measured at baseline, 4-, 8-, and 12- weeks post treatment entry. The patient evidenced a decrease in self-reported depression severity, measured with the Patient Health Questionnaire-9, from the moderate range at baseline (total score=12) to the mild range at week 12 (total score=8).

Likewise, the patient evidenced a decrease in self-reported anxiety severity, measured with the Generalized Anxiety Disorder-7 Scale, from the severe range at baseline (total score=15) to the mild range at week 12 (total score=5).

In addition to the UDS assay, adherence to buprenorphine was assessed via monthly telephone-based buprenorphine film counts over the 12-week study period, adapted from an empirically validated unannounced pill count procedure [22]. Trained research staff called the patient once per month to conduct a film count. Study staff completed the first film count 1-month post buprenorphine stabilization as determined by the study clinician. Each subsequent count allowed for an adherence score, ranging from 0 to 1, to be calculated by the ratio of films remaining relative to films prescribed, taking into account the number of films dispensed [23].

Opioid use frequency in the past 30 days was assessed monthly using a timeline follow-up interview [24]. At baseline, the patient reported 16 days of illicit opioid use in the previous 30 days. At 1-month follow-up, following his induction onto buprenorphine, he reported 0 days of opioid use in the previous 30 days, with 98% adherence to medication based on the unannounced film counts. At week 8, the patient reached stabilization on buprenorphine and reported 0 days of opioid use in the previous 30 days; however, this was inconsistent with his UDS, which was positive for opiates. Nevertheless, his unannounced film count revealed 80% medication adherence, which is clinically consistent with a temporary return to opioid use. At week 12, the patient’s opioid- and fentanyl-negative UDS was consistent with his self-reported abstinence from opioid use over the past 30 days, coupled with his film count which demonstrated 100% MOUD adherence. In addition, the patient’s craving for opioids, measured using a Visual Analog Scale (with a subjective craving intensity score ranging from 0 to 100), decreased by 98% between baseline and week 12.

The patient’s self-reported cigarette use was also captured during the timeline follow-up interview every 4 weeks, which showed a 75% reduction from 20 cigarettes per day at baseline, to 5 cigarettes per day at week 12. The patient was stable in his recovery from opioid and tobacco use disorder at the time of discharge from Pel-O. His discharge was prompted by the conclusion of the clinical trial intervention period, at which time he was referred, in accordance with his preferences, to another digital OUD care provider to continue his MOUD treatment regimen.

## Discussion

### Principal Findings

Despite living in a rural area where access to evidence-based treatment for OUD was scarce, the patient was able to receive care for co-occurring OUD and tobacco use disorder via a digital care modality. This promising treatment strategy enabled the patient to overcome barriers including transportation costs, financial burden, and stigma associated with receiving care and participating in UDS procedures. Although research to date has demonstrated the feasibility of digital treatment for OUD [10,20,25,26], evidence-based clinical guidelines to inform the practice of digital buprenorphine induction for individuals using highly potent synthetic opioids such as fentanyl are lacking [13,14]. As such, evidence from this case demonstrates the feasibility, safety, and potential efficacy of this highly scalable approach to making MOUD care accessible to individuals using fentanyl who would otherwise face numerous obstacles to receiving care.

Of note, consistent with research demonstrating that nonprescribed buprenorphine use is frequently observed among individuals who are attempting to taper themselves off opioids [27], the patient’s buprenorphine induction was preceded by nonprescribed buprenorphine use over a 2-week period, which likely contributed, in part, to his successful induction in the digital clinic setting. In addition, despite the challenges inherent to engaging and motivating individuals with OUD and co-occurring tobacco use disorder, the use of combination NRT coupled with behavioral treatment was effective in facilitating tobacco cessation simultaneously with recovery from OUD, which adds to the existing literature underscoring the need for concurrent treatment [28].

### The Patient’s Perspective

The major themes that emerged from the patient’s qualitative interview as well as interviews with other participants in the larger study are summarized in detail in the study by Monico et al (2025) [29]. The patient interviewed for this case report expressed that he felt cared for by his Pel-O physician and counselor:

[Prescriber] is a really, really good doctor. He really cares. I mean, he takes the time to sit down with you and ask you how you are.

The patient was most motivated by the digital doctor’s visits and counseling aspect of the Pel-O program, highlighting the contrast between his experience at Pel-O when compared with the difficulty of commuting at inconvenient times to a methadone clinic:

Well, I went to the methadone clinic about probably about eight years ago. And, you know, it kind of worked. But the way that they make you do it, you know, you have to drive 60 miles there every day, and pick it up at 6:00 in the morning and then drive 60 miles back every day. It just made it too hard.

The patient also expressed gratitude for the flexibility and convenience afforded by the digital care modality:

I actually loved it. I loved how convenient it was. You know, it made it where it was easier to talk to her, you know, to talk to my counselor because of how convenient it was. And it made me want to open up more, to [prescriber], to about, you know, and because it is so convenient. But I never thought I would be that honest with a counselor or a doctor, you know?

### Limitations

Several limitations of this report warrant comment, particularly in regard to the time-limited treatment duration imposed by the clinical trial context of this patient’s care. Specifically, the patient continued to use cannabis throughout his treatment, and the impact of this use on his short- and longer-term recovery from OUD warrants further examination in light of research examining the association between OUD treatment outcomes and cannabis use among individuals receiving MOUD [30]. Second, given his transition out of Pel-O approximately 2 months after reducing his use of tobacco, the stability of changes in his tobacco use is unknown. Future research elucidating the longer-term outcomes of individuals who concurrently discontinue or reduce their use of opioids and tobacco will have great use in identifying gaps in care where further innovation is needed to address polysubstance use.

### Conclusion

In conclusion, the case presented herein expands the growing body of literature supporting the safety and efficacy of MOUD delivered via telemedicine, extending the potential indication of this treatment modality to address polysubstance use and populations who present with recent use of potent synthetic opioids including fentanyl. The success of this case highlights the need for greater resource allocation to telehealth modalities for treating severe OUD, combining psychotherapy and MOUD to alleviate barriers associated with traditional, face-to-face treatment.

## Abbreviations

CBT: cognitive behavioral therapy
*DSM-5*: *Diagnostic and Statistical Manual of Mental Disorders (Fifth Edition)*
MET: motivational enhancement therapy
MOUD: medications for opioid use disorder
NRT: nicotine replacement therapy
OUD: opioid use disorder
Pel-O: Pelago Opioid
SUD: substance use disorder
THC: Tetrahydrocannabinol
UDS: urine drug screens

## Data Availability

The datasets generated or analyzed during this study are not publicly available due to the sensitive and highly confidential nature of substance use disorder data under 42 Code of Federal Regulations Part 2 but are available from the corresponding author on reasonable request subject to approval by the appropriate institutional review board and compliance with applicable redisclosure restrictions.

## References

[1] Shulman M, Wai JM, Nunes EV (2019). Buprenorphine treatment for opioid use disorder: an overview. CNS Drugs.

[2] Jones KF, O'Reilly Jacob M, Spetz J, Hailer L, Tierney M (2023). Eliminate the buprenorphine DEA X waiver: Justification using a policy analysis approach. J Nurs Scholarsh.

[3] Jones CM, Olsen Y, Ali MM, Sherry TB, Mcaninch J, Creedon T, Juliana P, Jacobus-Kantor L, Baillieu R, Diallo MM, Thomas A, Gandotra N, Sokolowska M, Ling S, Compton W (2023). Characteristics and prescribing patterns of clinicians waivered to prescribe buprenorphine for opioid use disorder before and after release of new practice guidelines. JAMA Health Forum.

[4] Chua K, Bicket MC, Bohnert ASB, Conti RM, Lagisetty P, Nguyen TD (2024). Buprenorphine dispensing after elimination of the waiver requirement. N Engl J Med.

[5] Lofwall MR, Fanucchi LC (2021). Long-acting buprenorphine injectables: Opportunity to improve opioid use disorder treatment among rural populations. Prev Med.

[6] Andrilla CHA, Moore TE, Patterson DG (2019). Overcoming barriers to prescribing buprenorphine for the treatment of opioid use disorder: recommendations from rural physicians. J Rural Health.

[7] Franz B, Dhanani LY, Hall OT, Brook DL, Simon JE, Miller WC (2023). Differences in buprenorphine prescribing readiness among primary care professionals with and without X-waiver training in the US. Harm Reduct J.

[8] Fenstemaker C, Abrams EA, Obringer B, King K, Dhanani LY, Franz B (2024). Primary care professionals' perspectives on tailoring buprenorphine training for rural practice. J Rural Health.

[9] Weintraub E, Seneviratne C, Anane J, Coble K, Magidson J, Kattakuzhy S, Greenblatt A, Welsh C, Pappas A, Ross TL, Belcher AM (2021). Mobile telemedicine for buprenorphine treatment in rural populations with opioid use disorder. JAMA Netw Open.

[10] Maricich YA, Xiong X, Gerwien R, Kuo A, Velez F, Imbert B, Boyer K, Luderer HF, Braun S, Williams K (2021). Real-world evidence for a prescription digital therapeutic to treat opioid use disorder. Curr Med Res Opin.

[11] Jones CM, Shoff C, Hodges K, Blanco C, Losby JL, Ling SM, Compton WM (2022). Receipt of telehealth services, receipt and retention of medications for opioid use disorder, and medically treated overdose among Medicare beneficiaries before and during the COVID-19 pandemic. JAMA Psychiatry.

[12] Srivastava A, Kahan M, Leece P, McAndrew A (2019). Buprenorphine unobserved "home" induction: a survey of Ontario's addiction physicians. Addict Sci Clin Pract.

[13] Samuels EA, Khatri UG, Snyder H, Wightman RS, Tofighi B, Krawczyk N (2022). Buprenorphine telehealth treatment initiation and follow-up during COVID-19. J Gen Intern Med.

[14] Gunderson EW, Wang X, Fiellin DA, Bryan B, Levin FR (2010). Unobserved versus observed office buprenorphine/naloxone induction: a pilot randomized clinical trial. Addict Behav.

[15] Silverstein SM, Daniulaityte R, Martins SS, Miller SC, Carlson RG (2019). "Everything is not right anymore": Buprenorphine experiences in an era of illicit fentanyl. Int J Drug Policy.

[16] Antoine D, Huhn AS, Strain EC, Turner G, Jardot J, Hammond AS, Dunn KE (2021). Method for successfully inducting individuals who use illicit fentanyl onto buprenorphine/naloxone. Am J Addict.

[17] Custodio L, Malone S, Bardo MT, Turner JR (2022). Nicotine and opioid co-dependence: Findings from bench research to clinical trials. Neurosci Biobehav Rev.

[18] Kathuria H, Seibert RG, Cobb V, Herbst N, Weinstein ZM, Gowarty M, Jhunjhunwala R, Helm ED, Wiener RS (2019). Perceived barriers to quitting cigarettes among hospitalized smokers with substance use disorders: A mixed methods study. Addict Behav.

[19] Vlad C, Arnsten JH, Nahvi S (2020). Achieving smoking cessation among persons with opioid use disorder. CNS Drugs.

[20] Monico LB, Eastlick M, Michero D, Pielsticker P, Glasner S (2024). Feasibility and acceptability of a novel digital therapeutic combining behavioral and pharmacological treatment for opioid use disorder. Digit Health.

[21] Mee-Lee D, Shulman GD, Fishman MJ, Gastfriend DR, Miller MM, Provence SM, Chevy Chase MD (2013). The ASAM criteria: Treatment for addictive, substance-related, and co-occurring conditions.

[22] Kalichman SC, Amaral CM, Stearns H, White D, Flanagan J, Pope H, Cherry C, Cain D, Eaton L, Kalichman MO (2007). Adherence to antiretroviral therapy assessed by unannounced pill counts conducted by telephone. J Gen Intern Med.

[23] Kalichman SC, Pellowski J, Kegler C, Cherry C, Kalichman MO (2015). Medication adherence in people dually treated for HIV infection and mental health conditions: test of the medications beliefs framework. J Behav Med.

[24] Hjorthøj Carsten Rygaard, Hjorthøj Anne Rygaard, Nordentoft M (2012). Validity of timeline follow-back for self-reported use of cannabis and other illicit substances--systematic review and meta-analysis. Addict Behav.

[25] Hailu R, Mehrotra A, Huskamp HA, Busch AB, Barnett ML (2023). Telemedicine use and quality of opioid use disorder treatment in the US during the COVID-19 pandemic. JAMA Netw Open.

[26] Meyer B, Utter G, Hillman C (2021). A personalized, interactive, cognitive behavioral therapy-based digital therapeutic (MODIA) for adjunctive treatment of opioid use disorder: development study. JMIR Ment Health.

[27] Lofwall MR, Walsh SL (2014). A review of buprenorphine diversion and misuse: the current evidence base and experiences from around the world. J Addict Med.

[28] Morris CD, Garver-Apgar CE (2020). Nicotine and opioids: a call for co-treatment as the standard of care. J Behav Health Serv Res.

[29] Monico LB, Eastlick M, Michero D, Pielsticker P, Glasner S (2025). Overcoming barriers to traditional care delivery and pharmacy challenges: a qualitative study of buprenorphine, telehealth, and a digital therapeutic for opioid use disorder. Subst Abuse Treat Prev Policy.

[30] Lake S, St Pierre M (2020). The relationship between cannabis use and patient outcomes in medication-based treatment of opioid use disorder: A systematic review. Clin Psychol Rev.

